# What are the differences between the three most used classifications for acute colonic diverticulitis? A comparative multicenter study

**DOI:** 10.1097/TA.0000000000004133

**Published:** 2023-09-04

**Authors:** Camilla Cremonini, Alan Biloslavo, Virna Robustelli, Sandro Giannessi, Simone Rossi Del Monte, Manuela Mastronardi, Serena Musetti, Silvia Strambi, Federico Coccolini, Massimo Chiarugi, Dario Tartaglia

**Affiliations:** From the General and Emergency Surgery Unit (C.C., S.M., S.S., F.C., M.C., D.T.), University of Pisa, Pisa, Italy; General Surgery Unit (A.B., M.M.), Cattinara University Hospital, Trieste, Italy; General Surgery Unit (V.R., S.G.), S. Jacopo Hospital, Pistoia, Italy; and General and Emergency Surgery (S.R.D.M.), San Filippo Neri Hospital, Rome, Italy.

**Keywords:** Acute diverticulitis, classifications, AAST, WSES, Hinchey, outcomes

## Abstract

**BACKGROUND:**

Acute left-sided colonic diverticulitis is one of the most common clinical conditions encountered by surgeons in the acute setting. Currently, the most popular classifications, based on radiological findings, are the modified Hinchey, American Association for the Surgery of Trauma (AAST), and World Society of Emergency Surgery (WSES) classifications. We hypothesize that all classifications are equivalent in predicting outcomes.

**METHODS:**

This is a retrospective study of 597 patients from four medical centers between 2014 and 2021. Based on clinical, radiological, and intraoperative findings, patients were graded according to the three classifications. Regression analysis and receiver operating characteristic curve analysis were used to compare six outcomes: need for intervention, complications, major complications (Clavien-Dindo >2), reintervention, hospital length of stay, and mortality.

**RESULTS:**

A total of 597 patients were included. Need for intervention, morbidity, and reintervention rates significantly increased with increasing AAST, modified Hinchey, and WSES grades. The area under the curve (AUC) for the need for intervention was 0.84 for AAST and 0.81 for modified Hinchey (*p* = 0.039). The AUC for major complications was 0.75 for modified Hinchey and 0.70 for WSES (*p* = 0.009). No differences were found between the three classifications when comparing AUCs for mortality, complications, and reintervention rates.

**CONCLUSION:**

The AAST, WSES, and modified Hinchey classifications are similar in predicting complications, reintervention, and mortality rates. American Association for the Surgery of Trauma and modified Hinchey scores result the most adequate for predicting the need for surgery and the occurrence of major complications.

**LEVEL OF EVIDENCE:**

Prognostic and Epidemiological; Level III.

## Abstract 2

Modified Hinchey, AAST, and WSES classifications are the most popular classifications for acute diverticulitis. 597 patients were retrospectively evalutated. AAST, WSES, and modified Hinchey classifications are similar in predicting complications, reintervention, and mortality.

## Graphical abstract

**Figure:**
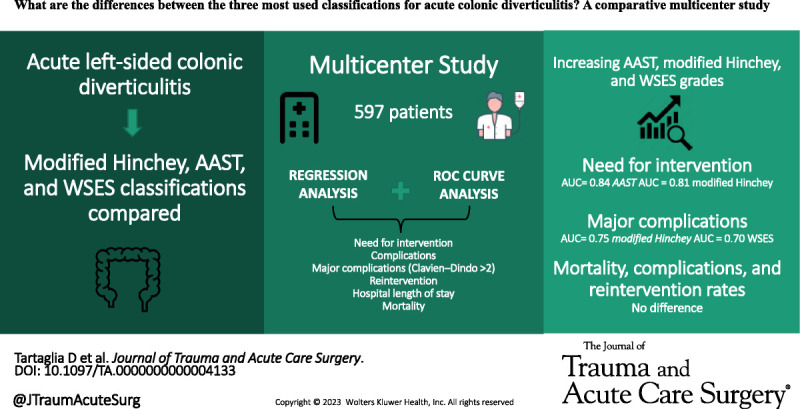

Acute colonic diverticulitis represents one of the most relevant diseases in emergency general surgery. Proper clinical assessment of the pathology can help surgeons to stratify patients who may require tailored medical and surgical strategies as an adequate patient categorization is associated with more appropriate clinical management. To objectively identify the severity and consequent patient outcomes, one of the tools at surgeons' disposal is classification systems. Several classifications have been proposed over the years for emergency diverticular disease.^1–5^ As the oldest and most used system, Hinchey classification takes into consideration both preoperative and intraoperative findings. Although there have been several attempts to modify it, mainly with the aim of discriminating stages I and II,^2^ there is still an objective difficulty in stratifying patients earlier. Therefore, the American Association for the Surgery of Trauma (AAST) and the World Society of Emergency Surgery (WSES) proposed two classifications that focus on patients' computed tomography (CT) images^5,6^ (Fig. 1). To date, no studies have specifically compared these three classification systems in terms of clinical utility and outcomes, although there has been an attempt to compare the AAST and Hinchey classifications; however, the latter still represents the established scoring system for acute diverticulitis.^7^ The aim of the present study was to compare AAST, WSES, and modified Hinchey classifications of acute colonic diverticulitis in terms of their ability to predict the need for procedural intervention, mortality, hospital length of stay (HLOS), intensive care unit (ICU) admission, and complications. We hypothesized that there are no differences among these three main classifications regarding these outcomes.

**Figure 1:**
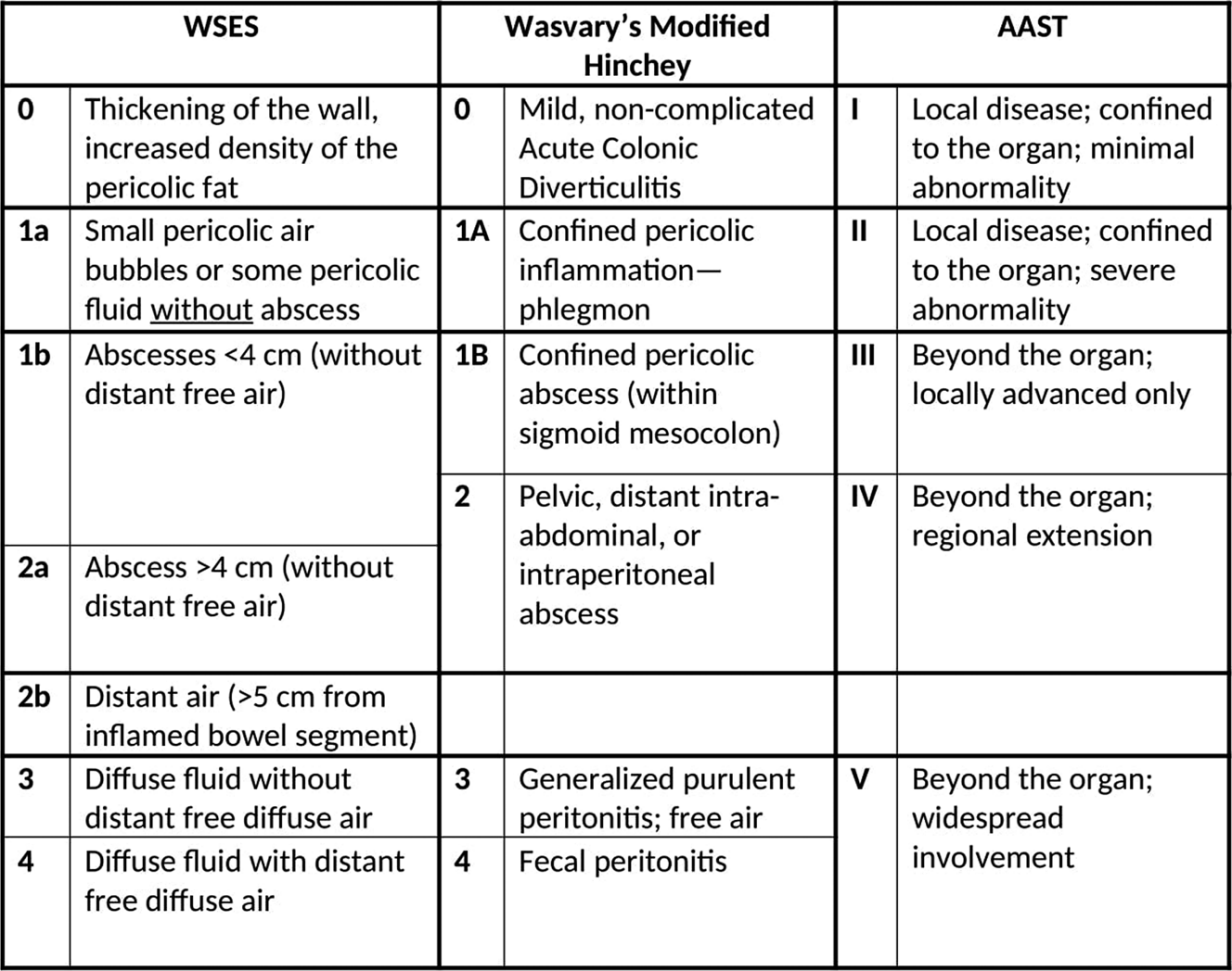
Three most used classifications for diverticular disease.

## METHODS

We retrospectively evaluated the charts of patients admitted with acute colonic diverticulitis at four large academic medical centers. The study population was derived from a consecutive patient sample gathered from 2014 to 2021. Adult patients (older than 18 years) were included in the study if they were admitted with left-sided acute colonic diverticulitis. The present study has been registered at ClinicalTrial.org with the number NCT04596280. Having CT images obtained at the time of admission was an essential inclusion criterion. Each admission was inspected via chart review. Demographic and clinical data were collected, including age, gender, comorbidities (hypertension, cardiopathy, smoking status, diabetes mellitus, chronic obstructive pulmonary disease), and medication status (steroids, anticoagulation, and immunosuppression). The term “cardiopathy” included all the heterogeneous diseases or disorders of the heart that could potentially lead to heart failure or severe arrhythmias. In those patients presenting with either purulent or fecal diffuse peritonitis, suture of the skin was initially avoided and delayed in the following postoperative days. The primary outcome was the need for procedural intervention (operation or percutaneous drainage). Secondary outcomes included ICU admission, in-hospital complications (incisional surgical site infection, renal failure, deep vein thrombosis/pulmonary embolism, urinary tract infection, major adverse cardiac event, need for mechanical ventilation), reintervention rate, HLOS, and in-hospital mortality. Both superficial and deep incisional surgical site infections were taken into consideration. Reinterventions were considered surgical operations required after the initial surgical treatment. Computed tomography images were examined by experienced radiologist reviewers from each participating center, and each scan was assigned an AAST grade, a WSES grade, and a modified Hinchey grade (Fig. 1). Radiologists were blinded to the interventions and outcomes prior to reviewing CT scans. The study was conducted according to the STROBE guidelines^8^ (Supplemental Digital Content, http://links.lww.com/TA/D242). Data collection and analysis were performed according to the institutional guidelines and to the ethical standards of the Helsinki Declaration.

### Statistics

A computerized spreadsheet (Microsoft Excel 2013; Microsoft Corporation; Redmond, WA) was used for data collection. Analyses were performed using SPSS version 23 (SPSS Inc., Chicago, IL). Descriptive statistics were calculated for all clinical variables; categorical variables are presented as n (%), and continuous variables are represented as median (interquartile range [IQR]). Shapiro-Wilk normality test was checked for continuous variables. Univariate analysis of unadjusted outcomes was performed using Pearson's χ^2^ test or Fisher's exact test for categorical variables and the Kruskal-Wallis test for continuous variables. Adjusted outcomes were compared via logistic regression (need for procedure, morbidity, major complications, reinterventions, and mortality) and linear regression (HLOS) analysis. Receiver operating characteristic (ROC) curves were created, controlling for demographic and comorbidity risks. Nonparametric comparison of the modified Hinchey, AAST, and WSES ROC curves were compared, adjusting for covariates. Statistical significance was set at *p* = 0.05.

## RESULTS

A total of 597 patients were enrolled in the study. Demographic and clinical characteristics are presented in Table 1. The mean age was 65 years, and 57% of the patients were female. Median body mass index was 25.1 kg/m^2^, and 29% of cases had ASA score ≥3. Thirty-five percent of patients had previous history of acute diverticulitis. The most common medical comorbidities were hypertension (41%), cardiopathy (19%), and current smoking (16%). Conservative management was attempted in 41% of cases (Table 2). In the remaining 59% of patients, several interventions were performed, and a percutaneous drain was placed in 3% of these patients. In-hospital outcomes are described in Table 2.

**TABLE 1:** Demographics and Comorbidities

		Total Population = 597
Age: median (IQR), y		65 (55–77)
Age ≥70 y		251 (42%)
Gender		
Female		338 (57%)
Male		259 (43%)
BMI (median, IQR)		25.1 (22.7–28.2)
ASA ≥3*		170 (29%)
Previous diverticulitis		206 (35%)
Comorbidities		
Cardiopathy		116 (19%)
Vascular disease		91 (15%)
Hypertension		246 (41%)
COPD		77 (13%)
Diabetes		62 (10%)
Smoking		97 (16%)

Categorical variables are presented as n (%); continuous variables are presented as median with IQR.

BMI, body mass index; COPD, chronic obstructive pulmonary disease.

*148 missing values.

**TABLE 2:** Interventions, Outcomes, and Complications

		Total Population = 597
Therapy		
Conservative management		243 (41%)
Intervention		354 (59%)
Type of intervention*		
Hartmann resection		92 (26%)
Resection-anastomosis, diversion		62 (18%)
Resection-anastomosis, no diversion		159 (45%)
Laparoscopic peritoneal lavage		24 (7%)
Percutaneous drainage		12 (3%)
Other**		5 (1%)
Laparoscopic†		167 (49%)
Conversion‡		48 (29%)
HLOS (median, IQR)		9 (6–14)
PLOS (median, IQR)		9 (7–13)
Complications		163 (27%)
Major complications (C-D ≥ 3)		66 (11%)
Type of complications		
Incisional surgical site infection§		19 (9%)
Pneumonia		16 (3%)
Sepsis		31 (5%)
Transfusions		30 (5%)
Cardiac event		23 (4%)
Pulmonary embolism		3 (0.5%)
Respiratory failure		27 (5%)
Renal failure		12 (2%)
Reintervention		45 (8%)
Mortality		31 (5%)

Categorical variables are presented as n (%); continuous variables are presented as median with IQR.

*% of overall interventions: n = 354)

**Diversion without resection, total colectomy, dead as damage-control surgery or unknown.

†% of overall surgeries: n = 342)

‡% of overall laparoscopic approaches: n = 167)

§Superficial and Deep incisional surgical site infection; the % was calculated over the population of patients with a laparotomic incision (total number = 223).

PLOS, postoperative length of stay; C-D, Clavien-Dindo.

Unadjusted outcomes for modified Hinchey, AAST, and WSES classifications are presented in Table 3. In general, the need for intervention significantly increased as modified Hinchey, AAST, and WSES grade increased (*p* < 0.001). A similar trend was observed for other outcomes, like morbidity (*p* < 0.001), major complications (*p* < 0.001), and reintervention rates (*p* < 0.001). Mortality increased significantly with increased modified Hinchey (*p* < 0.001) and AAST (*p* = 0.005) grade, while this trend was not significant for WSES grade (*p* = 0.06) (Table 3). The mean LOS was 9 days (IQR, 6–14 days), showing a significant increased tendency (*p* < 0.001).

**TABLE 3:** Unadjusted Outcomes for Hinchey, AAST, and WSES Classifications (Univariate Analysis)

	Total, N = 597	Hinchey 1a, n = 269	Hinchey 1b, n = 82	Hinchey 2, n = 94	Hinchey 3, n = 102	Hinchey 4, n = 50	*p*
Intervention	354 (59%)	88 (33%)	42 (51%)	73 (78%)	101 (99%)	50 (100%)	**<0.001**
Complication	163 (27%)	31 (12%)	18 (22%)	32 (34%)	44 (43%)	38 (76%)	**<0.001**
Major complication	66 (11%)	8 (3%)	6 (7%)	15 (16%)	18 (18%)	19 (38%)	**<0.001**
Reintervention	45 (8%)	6 (2%)	5 (6%)	11 (12%)	8 (8%)	15 (30%)	**<0.001**
Mortality	31 (5%)	8 (3%)	3 (4%)	3 (3%)	7 (7%)	10 (20%)	**<0.001**
HLOS	9 (6–14)	7 (5–10)	9 (6–14)	10 (8–16)	11 (9–14)	13 (9–22)	**<0.001**

	Total, N = 597	AAST 1, n = 242	AAST 2, n = 67	AAST 3, n = 97	AAST 4, n = 40	AAST 5, n = 151	*p*
Intervention	354 (59%)	67 (28%)	32 (48%)	75 (77%)	32 (80%)	148 (98%)	**<0.001**
Complication	163 (27%)	24 (10%)	10 (15%)	36 (37%)	13 (33%)	80 (53%)	**<0.001**
Major complication	66 (11%)	7 (3%)	2 (3%)	16 (17%)	6 (15%)	35 (23%)	**<0.001**
Reintervention	45 (8%)	5 (2%)	3 (5%)	10 (10%)	5 (13%)	22 (15%)	**<0.001**
Mortality	31 (5%)	6 (3%)	3 (5%)	3 (3%)	2 (5%)	17 (11%)	**0.005**
HLOS	9 (6–14)	7 (5–10)	8 (5–13)	11 (7–17)	10 (7–19)	11 (9–16)	**<0.001**

	Total, N = 597	WSES 0, n = 206	WSES Ia, n = 93	WSES Ib, n = 59	WSES IIa, n = 70	WSES IIb, n = 36	WSES III, n = 31	WSES IV, n = 102	*p*
Intervention	354 (59%)	57 (28%)	45 (48%)	34 (58%)	57 (81%)	32 (89%)	29 (94%)	100 (98%)	**<0.001**
Complication	163 (27%)	19 (9%)	19 (20%)	16 (27%)	26 (37%)	14 (39%)	15 (48%)	54 (53%)	**<0.001**
Major complication	66 (11%)	7 (3%)	4 (4%)	9 (15%)	11 (16%)	7 (19%)	6 (19%)	22 (22%)	**<0.001**
Reintervention	45 (8%)	4 (2%)	2 (2%)	8 (14%)	8 (11%)	3 (8%)	4 (13%)	16 (16%)	**<0.001**
Mortality	31 (5%)	6 (3%)	3 (3%)	3 (5%)	3 (4%)	3 (8%)	1 (3%)	12 (12%)	0.060
HLOS	9 (6–14)	7 (5–10)	8 (6–10)	10 (7–16)	10 (8–19)	11 (8–13)	21 (13–32)	15 (10–23)	**<0.001**

Categorical variables are presented as n (%); continuous variables are presented as median with IQR.

Regression analysis comparing adjusted outcomes revealed significantly increased odds with increasing modified Hinchey, AAST, and WSES grade (Table 4). The odds of needing operation or intervention were 2.16, 7.15, and 207.73 for modified Hinchey Grades 1b, 2, and 3, respectively, when referring to Hinchey Grade 1. Furthermore, AAST Grades 2, 3, 4, and 5, relative to Grade 1 (OR: 2.33, 8.90, 10.45, 128.86, respectively), and WSES 1b, 2a, 2b, 3, and 4, relative to Grade 1a (OR: 2.45, 3.55, 11.46, 20.91, 37.90, 130.7, respectively), were risk factors for intervention. A similar trend was observed for other outcomes, including complications, major complications, and reintervention (Table 4). The highest grade of every classification (modified Hinchey score 4, AAST Grade 5, and WSES class IV) were risk factors for mortality (OR: 8.51, 5.35, and 4.92 respectively), while lower grades were not significantly correlated with mortality rate. Linear regression analysis for HLOS revealed a significant increase in LOS for modified Hinchey Grades 2, 3, and 4. The same increase in LOS was identified in the linear regression analysis for AAST and WSES grades (Table 4).

**TABLE 4:** Adjusted Scoring Outcomes for Hinchey, AAST and WSES Classifications (Regression Analysis)

	Hinchey 1a (n = 269)	Hinchey 1b (n = 82)		Hinchey 2 (n = 94)		Hinchey 3 (n = 102)		Hinchey 4 (n = 50)	
		OR	95% CI	OR	95% CI	OR	95% CI	OR	95% CI
Intervention	REF	**2.16**	**1.30–3.57**	**7.15**	**4.13–12.37**	**207.73**	**28.50–1513.72**	—	**—**
Complication	REF	**2.16**	**1.14–4.10**	**3.96**	**2.25–6.99**	**5.82**	**3.39–10.01**	**24.31**	**11.49–51.42**
Major complication	REF	2.57	0.87–7.65	**6.19**	**2.53–15.14**	**6.99**	**2.93–16.66**	**19.99**	**8.08–49.48**
Reintervention	REF	2.85	0.84–9.58	**5.81**	**2.08–16.18**	**3.73**	**2.08–16.19**	**18.78**	**6.84–51.59**
Mortality	REF	1.24	0.32–4.78	1.07	0.28–4.14	2.40	0.84–6.81	**8.16**	**3–04-21.89**
HLOS	REF	3.14	0.93–5.34	**6.16**	**4.09–8.23**	**4.20**	**2.16–6.23**	**8.51**	**5.83–11.19**

	AAST 1 (n = 242)	AAST 2 (n = 67)		AAST 3 (n = 97)		AAST 4 (n = 40)		AAST 5 (n = 151)	
		OR	95% CI	OR	95% CI	OR	95% CI	OR	95% CI
Intervention	REF	**2.33**	**1.37–4.16**	**8.90**	**5.12–15.47**	**10.45**	**4.58–23.82**	**128.86**	**39.70–418.16**
Complication	REF	1.59	0.72–3.52	**5.36**	**2.97–9.66**	**4.37**	**1.99–9.58**	**10.23**	**6.03–17.37**
Major complication	REF	1.03	0.21–5.09	**6.63**	**2.63–16.69**	**5.92**	**1.87–18.67**	**10.12**	**4.36–23.49**
Reintervention	REF	2.22	0.52–9.55	**5.45**	**1.81–16.38**	**6.77**	**1.86–24.84**	**8.08**	**2.99–21.85**
Mortality	REF	1.84	0.45–7.57	1.25	0.31–5.12	2.07	0.40–10.64	**4.99**	**1.92–12.96**
HLOS	REF	1.38	−1.00-3.78	**5.97**	**3.88–8.07**	**5.76**	**2.72–8.80**	**5.35**	**3.53–7.16**

	WSES 0 (n = 206)	WSES Ia (n = 93)		WSES Ib (n = 59)		WSES IIa (n = 70)		WSES IIb (n = 36)		WSES III (n = 31)		WSES IV (n = 102)	
		OR	95% CI	OR	95% CI	OR	95% CI	OR	95% CI	OR	95% CI	OR	95% CI
Intervention	REF	**2.45**	**1.47–4.07**	**3.55**	**1.95–6.45**	**11.46**	**5.83–22-51**	**20.91**	**7.07–61.78**	**37.90**	**8.75–164.04**	**130.70**	**31.19–547.58**
Complication	REF	**2.52**	**1.27–5.04**	**3.66**	**1.74–7.69**	**5.86**	**2.95–11.44**	**6.26**	**2.76–14.21**	**9.23**	**3.95–21.54**	**11.07**	**6.01–20.40**
Major complication	REF	1.27	0.36–4.47	**5.12**	**1.82–14.40**	**5.30**	**1.96–14.28**	**6.86**	**2.24–20.98**	**6.82**	**2.12–21.92**	**7.81**	**3.21–19-02**
Reintervention	REF	1.110	0.20–6.17	**7.92**	**2.29–27.34**	**6.52**	**1.89–22.37**	**4.59**	**0.98–21.45**	**7.48**	**1.76–31.67**	**9.39**	**3.05–28.91**
Mortality	REF	1.11	0.27–4.54	1.79	0.43–7.37	1.49	0.36–6.13	3.03	0.72–12.71	1.11	0.13–9.55	**4.44**	**1.62–12.22**
HLOS	REF	0.79	−1.37 to 2.95	**4.77**	**2.20–7.34**	**6.84**	**4.41–9.27**	**3.91**	**0.82–7.00**	**8.96**	**5.50–12.41**	**4.92**	**2.82–7.03**

Values with *p* < 0.005 in bold.

**Figure 2:**
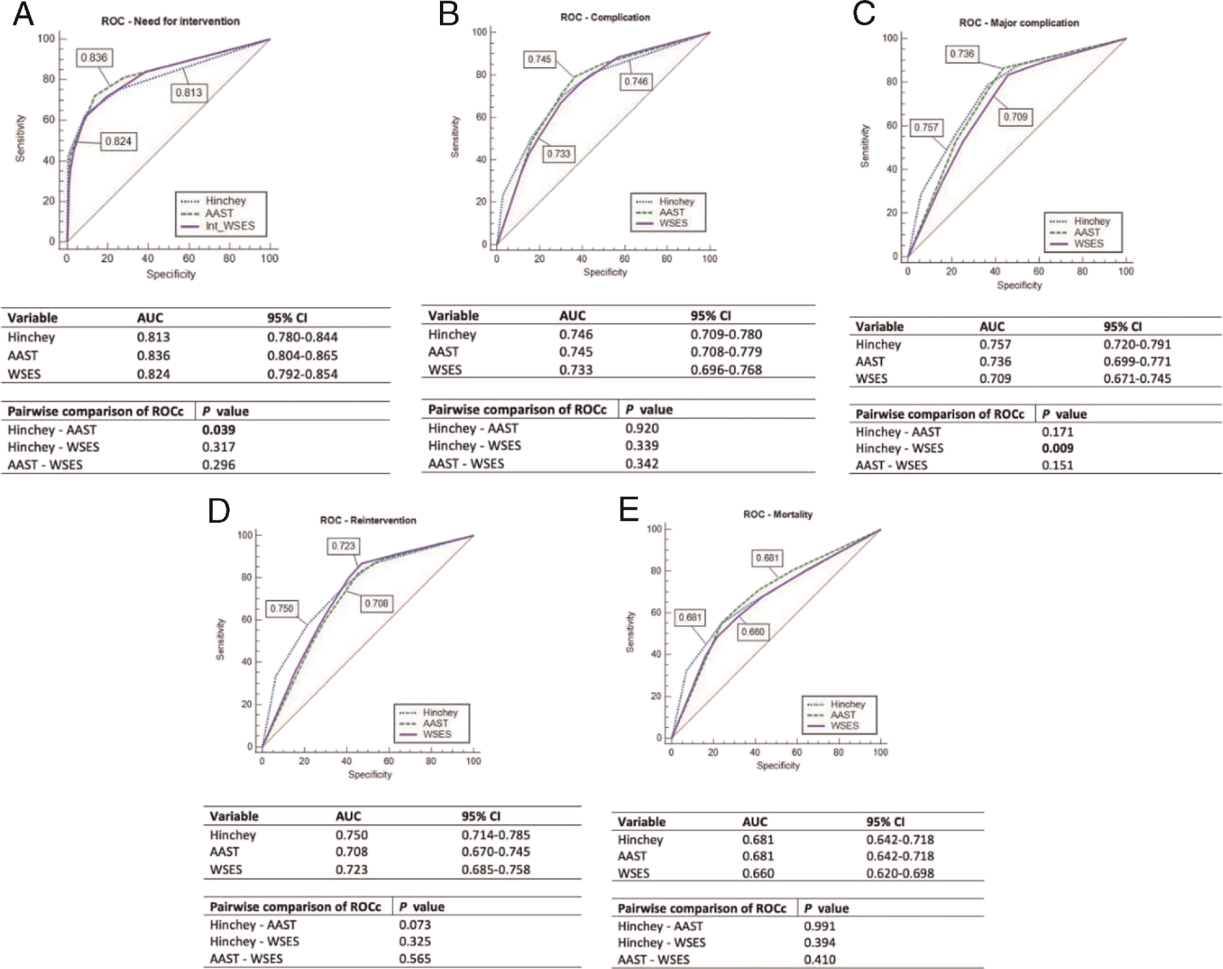
ROC curves. (*A*) Need for intervention; (*B*): Complication; (*C*) Major complication; (*D*) Reintervention; (*E*) Mortality.

Receiver operating characteristic curves were generated for the outcomes “need for intervention,” “morbidity,” “major complications,” “reintervention,” and “mortality,” with binary variables (Fig. 2). The c-statistics for predicting the need for intervention for the modified Hinchey, AAST, and WSES grades were 0.813, 0.836, and 0.824, respectively (Hinchey vs. AAST: *p* = 0.039). The c-statistics for major complications for the modified Hinchey, AAST, and WSES grades were 0.757, 0.736, and 0.709, respectively (Hinchey vs. WSES *p* = 0.009). The area under the ROC curve for the three classifications did not differ significantly for morbidity (0.746 vs. 0.745 vs. 0.733), reinterventions (0.750 vs. 0.708 vs. 0.723), or mortality (0.681 vs. 0.681 vs. 0.660).

## DISCUSSION

According to our analysis, the three selected classification systems were effective for predicting major outcomes including intervention, morbidity, reintervention, and HLOS; higher scores were significantly related to increased complication rates (in general and for major complications alone) and need for surgery or reintervention. In contrast, only high grades (Hinchey Grade 4, AAST Grade 5, and WSES Grade 4) were independent risk factors for increased mortality. The modified Hinchey, AAST, and WSES classifications did not differ significantly in their ability to predict morbidity, reinterventions, or mortality. AAST grading was more accurate than the modified Hinchey and WSES classifications when evaluating the need for intervention (c-statistics: AAST = 0.836 vs. WSES = 0.824 vs. modified Hinchey = 0.813), and AAST was more accurate than modified Hinchey for predicting major complications (c-statistics: modified Hinchey = 0.757 vs. AAST = 0.736 vs. WSES = 0.709).

This study aimed to compare the accuracy of the most frequently used classifications for acute diverticulitis. Few such comparisons are reported in the current literature. To our knowledge, only one study compared grading systems. Erbesole et al. retrospectively analyzed 129 patients with acute diverticulitis and compared the modified Hinchey classification and the AAST grading scale. They found that the need for operation c-statistics were 0.80 for Hinchey and 0.83 for AAST (*p* = 0.35), and the complication c-statistics curve was 0.83 for AAST and 0.80 for Hinchey (*p* = 0.33). In contrast, the AAST and Hinchey scores were less predictive for ICU admission, readmission, and mortality, with c-statistics less than 0.80. Therefore, the authors concluded that the AAST classification for acute diverticulitis is equivalent to the modified Hinchey classification for predicting procedural interventions and complications, but the AAST system is preferred to Hinchey because it can be applied preoperatively.^7^ However, the study was unable to achieve a definitive conclusion, as the sample size was relatively small and underpowered by the fact that it was conducted in a single-center setting.

From the first classification published by Hinchey et al.,^1^ the increasing number of classifications proposed for acute diverticulitis has created a *Babel* among specialists.^2,5,9–14^ In a review published in 2012, Klarenbeek et al.^14^ proposed that a proper classification system could improve mutual communication between doctors from different specialties and support clinical decision making.

The principal limitation of the original Hinchey score is its lack of application in the preoperative setting as this system grades only intraoperative findings. The effort to improve the Hinchey classification with further sub-classifications has not addressed this weakness. Moreover, the modified classification still has important “gray” areas that are not adequately represented by the clinical and radiological findings. For example, the presence of bubbles close to the colon or far from the diverticular site, in the absence of significant peritoneal collections, would be difficult to properly classify with the Hinchey system.^15^ The AAST and WSES classification systems aimed to overcome the lack of preoperative specificity of the modified Hinchey classification by stratifying CT findings. Their spread and progressive use in the literature has raised the important question of these two scales could improve risk stratification and patient management. As stated by the European Society of Coloproctology, no CT scan-based classification is superior to others as a diagnostic tool for acute diverticulitis, and each center should adopt their preferred classification, in accordance with the radiologist personnel.^16^

This study has several limitations. First, some in-hospital items and follow-up criteria, such as the need for ICU admission, recurrence rate, and need for elective surgery, were not considered. However, we speculate that the addition of these outcomes would have reduced the significance of the results, as these outcomes can be potentially influenced by other clinical variables. Furthermore, the cohort of patients was selected from several centers, and clinical management practices may vary from hospital to hospital. Similarly, we did not consider patients who were discharged from general medicine departments with presumably mild disease. However, the strength of this study is the sample size, that was considerable. As a multicenter study, we were able to enroll a large enough patient population to adequately evaluate the secondary outcomes.

In conclusion, AAST, WSES, and modified Hinchey classifications are adequate tools in the management of left-sided acute colonic diverticulitis. All three classification systems are equally effective for predicting outcomes, like complications, need for reintervention, and mortality. American Association for the Surgery of Trauma and modified Hinchey scores result the most adequate for predicting the need for surgery and the occurrence of major complications.

## Supplementary Material

SUPPLEMENTARY MATERIAL

## References

[1] Hinchey E Schaal P Richards G. Treatment of perforated diverticular disease of the colon. *Adv Surg*. 1978;12:85–109. 735943

[2] Wasvary H Turfah F Kadro O Beauregard W. Same hospitalization resection for acute diverticulitis. *Am Surg*. 1999;65(7):632–635; discussion 636. 10399971

[3] Ambrosetti P Grossholz M Becker C Terrier F Morel P. Computed tomography in acute left colonic diverticulitis. *Br J Surg*. 1997;84(4):532–534. 9112910 10.1046/j.1365-2168.1997.02576.x

[4] Shafi S Aboutanos M Brown CV Ciesla D Cohen MJ Crandall ML, . Measuring anatomic severity of disease in emergency general surgery. *J Trauma Acute Care Surg*. 2014;76(3):884–887. 24553565 10.1097/TA.0b013e3182aafdba

[5] Sartelli M Moore FA Ansaloni L Di Saverio S Coccolini F Griffiths EA, . A proposal for a CT driven classification of left colon acute diverticulitis. *World J Emerg Surg*. 2015;10:3. 25972914 10.1186/1749-7922-10-3 PMC4429354

[6] Emergency General Surgery Anatomic Grading Scales. The American Association for the Surgery of Trauma. Available at: http://www.aast.org/emergency-general-surgery-anatomic-grading-scales. Accessed July 12, 2019.

[7] Ebersole J Medvecz AJ Connolly C Sborov K Matevish L Wile G, . Comparison of American Association for the Surgery of Trauma grading scale with modified Hinchey classification in acute colonic diverticulitis: a pilot study. *J Trauma Acute Care Surg*. 2020;88(6):770–775. 32118825 10.1097/TA.0000000000002650

[8] Von Elm E Altman DG Egger M Pocock SJ Gøtzsche PC Vandenbroucke JP, STROBE initiative. The strengthening the reporting of observational studies in epidemiology (STROBE) statement: guidelines for reporting observational studies. *Lancet*. 2007;370(9596):1453–1457. 18064739 10.1016/S0140-6736(07)61602-X

[9] Hansen O Graupe F Stock W. [Prognostic factors in perforating diverticulitis of the large intestine] [in German]. *Chirurg*. 1998;69:443–449. 9612631 10.1007/s001040050436

[10] Ambrosetti P Becker C Terrier F. Colonic diverticulitis: impact of imaging on surgical management—a prospective study of 542 patients. *Eur Radiol*. 2002;12:1145–1149. 11976860 10.1007/s00330-001-1143-y

[11] Siewert JR Huber FT Brune IB. Early elective surgery of acute diverticulitis of the colon. *Chirurg*. 1995;66(12):1182–1189. 8582161

[12] Köhler L Sauerland S Neugebauer E. Diagnosis and treatment of diverticular disease: results of a consensus development conference. The scientific Committee of the European Association for endoscopic surgery. *Surg Endosc*. 1999;13:430–436. 10094765 10.1007/s004649901007

[13] Kaiser AM Jiang JK Lake JP Ault G Artinyan A Gonzalez-Ruiz C, . The management of complicated diverticulitis and the role of computed tomography. *Am J Gastroenterol*. 2005;100:910–917. 15784040 10.1111/j.1572-0241.2005.41154.x

[14] Klarenbeek BR de Korte N van der Peet DL Cuesta MA. Review of current classifications for diverticular disease and a translation into clinical practice. *Int J Colorectal Dis*. 2012;27(2):207–214. 21928041 10.1007/s00384-011-1314-5 PMC3267934

[15] Biondo S Lopez Borao J Millan M Kreisler E Jaurrieta E. Current status of the treatment of acute colonic diverticulitis: a systematic review. *Colorectal Dis*. 2012;14:e1–e11. 21848896 10.1111/j.1463-1318.2011.02766.x

[16] Schultz JK Azhar N Binda GA Barbara G Biondo S Boermeester MA, . European Society of Coloproctology: guidelines for the management of diverticular disease of the colon. *Colorectal Dis*. 2020;22(Suppl 2):5–28. 10.1111/codi.15140 32638537

